# A Selection Index for Gene Expression Evolution and Its Application to the Divergence between Humans and Chimpanzees

**DOI:** 10.1371/journal.pone.0034935

**Published:** 2012-04-18

**Authors:** Maria Warnefors, Adam Eyre-Walker

**Affiliations:** School of Life Sciences, University of Sussex, Brighton, United Kingdom; UC Santa Barbara, United States of America

## Abstract

The importance of gene regulation in animal evolution is a matter of long-standing interest, but measuring the impact of selection on gene expression has proven a challenge. Here, we propose a selection index of gene expression as a straightforward method for assessing the mode and strength of selection operating on gene expression levels. The index is based on the widely used McDonald-Kreitman test and requires the estimation of four quantities: the within-species and between-species expression variances as well as the sequence heterozygosity and divergence of neutrally evolving sequences. We apply the method to data from human and chimpanzee lymphoblastoid cell lines and show that gene expression is in general under strong stabilizing selection. We also demonstrate how the same framework can be used to estimate the proportion of adaptive gene expression evolution.

## Introduction

It has long been suggested that the phenotypic divergence between species is often due to alterations in gene expression [1]–[3]. It is therefore of great interest to investigate the selection pressures that shape gene expression evolution. If the regulatory regions are already known, a number of sequence analysis tools can be used to test for positive and negative selection acting on the relevant sequences [4]–[7]; however, such information is scarce. While expression quantitative trait loci (eQTLs) have been used to detect very recent cases of positive selection [8], the use of sequence analysis methods on a larger scale generally relies on assumptions regarding which sequences are involved in regulation [9]–[13] and will therefore exclude currently unidentified regulators, such as many distant-acting elements, in spite of their potentially substantial contribution to gene regulation [14]. Furthermore, the studied sequences may experience selection due to other reasons, which could mistakenly be attributed to gene regulation.

A more desirable solution would therefore be to infer selection directly from gene expression data without requiring knowledge of regulatory sequences. Much effort has been made to investigate the evolutionary dynamics of gene expression and identify expression shifts that may be due to adaptive evolution [15]–[19], but the interpretation of these results is not straightforward as our limited knowledge of gene expression evolution makes it difficult to establish a suitable null model against which observations can be evaluated. To overcome this issue, Fraser et al. [20] used the prediction that eQTLs affecting neutrally evolving genes would not tend to change expression in a specific direction to search for positively selected genes in mice, however the method requires the investigated lineages to be able to produce hybrid offspring and is therefore unsuitable for most comparisons between species. A second option has been to estimate the magnitude of gene expression divergence under neutral evolution based on the mutational variance [21] or the mutational heritability [22], but to directly estimate these quantities from mutation accumulation experiments is only feasible for species with short generation times that can be reared under laboratory conditions [23], [24]. For other species, such as humans and chimpanzees, it has been suggested that expressed pseudogenes could serve as a neutral standard [25], but it is not clear whether they fulfil the requirement of being non-functional [26] and they are not common.

The alternative to estimating the rate of neutral gene expression evolution experimentally is to develop a null hypothesis based on theoretical models. Both neutral models, i.e., where gene expression divergence increases linearly with time [27] and models where the increase in expression divergence is curbed by stabilising selection [28] have been proposed. While these models may appear mutually exclusive, it may rather be that they represent different evolutionary phases. Studies of expression divergence in seven *Drosophila* species indicate that gene expression divergence increases rapidly following speciation, but that the rate of the increase soon tapers off [28]. Thus gene expression evolution in very closely related species may be best approximated by a neutral model [27], whereas models that rely on expression optima [28] may be more appropriate for more diverged species.

Here we present a selection index of gene expression, which can be used to evaluate the selective forces that shape gene expression in a pair of species. The method is an extension of the McDonald-Kreitman framework, which is frequently used to estimate selection acting on DNA sequences [29]–[31]. When the selection index is close to zero, it indicates that gene expression evolves neutrally, while negative values indicate stabilising selection and positive values indicate directional selection. In the latter case, it is furthermore possible to estimate the proportion of gene expression evolution that is adaptive.

## Materials and Methods

In this paper, we describe a gene expression selection index, based on the McDonald-Kreitman (MK) test, which was developed for sequence data. In the MK test the numbers of synonymous (*P_s_*) and non-synonymous (*P_n_*) polymorphisms are compared to the numbers of synonymous (*D_s_*) and non-synonymous (*D_n_*) substitutions. Under a neutral model in which mutations at synonymous sites are neutral and mutations at non-synonymous sites are neutral or strongly deleterious, *D_n_/D_s_* = *P_n_/P_s_*. In contrast if some non-synonymous mutations are advantageous *D_n_/D_s_*>*P_n_/P_s_*, and if some are slightly deleterious *D_n_/D_s_*<*P_n_/P_s_* [29].

We can formulate a selection index for gene expression divergence as follows: Let us assume that mutations that affect gene expression are either neutral or strongly deleterious, and that a proportion, *f*, of mutations is neutral. Let us also assume that the evolution of gene expression over a short time follows that of a random walk, where expression is measured as the logarithm of the abundance. If *X(t)* is the expression level at time *t*, then
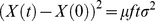
(1)where *μ* is the mutation rate and *σ^2^* is the increase of gene expression per neutral mutation [32]. Hence the squared difference in expression between two individuals, be they of the same or different species is
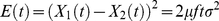
(2)


The squared difference is expected to increase linearly with time, *i.e.* the variance in gene expression between individuals is expected to increase linearly with time [32], [33]. This is expected to be true over the shorter time scale, but there will eventually be limits as to how high or low expression can evolve [28].

**Figure 1:**
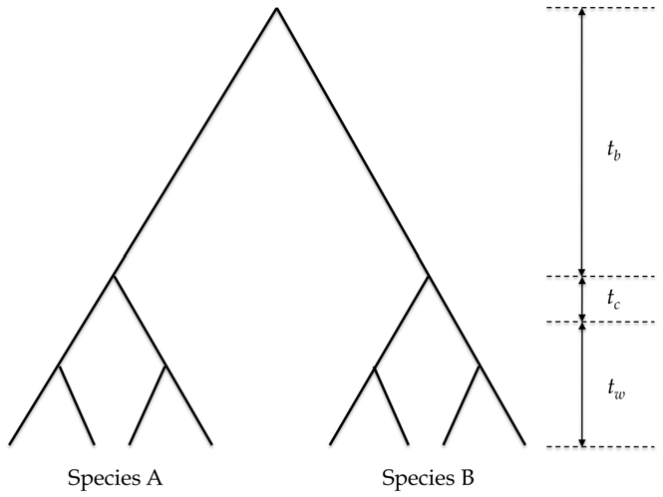
Tree illustrating the time between the most recent common ancestors of each species (*t_b_*), the expected time to coalescence for two randomly chosen lineages within a given species (*t_w_*) and the difference between *t_w_* and the time at which all lineages coalesce (*t_c_*).

Let us split the divergence between the two individuals into three time periods: *t_b_*, the time between the most recent common ancestors in each species for the locus in question; *t_wi_*, the expected time to coalescence for two randomly chosen lineages in species *i*, and *t_ci_*, the difference between *t_wi_* and the time at which all lineages coalesce (Figure 1). For a recombining sequence each of these times will be the average across sites within the locus in question. If mutations are strongly deleterious or neutral, then the sequence divergence between individuals is linearly related to the time that separates them
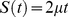
(3)so the divergence between species, *S_b_*, is expected to equal *S(t_b_)* and the divergence between individuals of the same species, *S_w_*, is expected to be *S(t_w_)*.

We can make a similar argument for expression divergence: The expected expression divergence between species, *E_b_*, is therefore expected to be equal to *E(t_b_)* and the average expression divergence between pairs of individuals within a species, *E_w_*, is expected to be *E(t_w_)*. Let us also define *E_c_* = *E(t_c_)*. Hence we expect under strict neutrality to have *E_b_/E_w_* = *S_b_/S_w_*. This may be rearranged analogously to the MK test above: *E_b_/S_b_* = *E_w_/S_w_*, to give the selection index, which is similar to the fixation index that has been proposed for nucleotide sequences [34]–[36]:
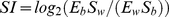
(4)


We need to estimate the variance in expression between species (*E_b_*) and between individuals within a species (*E_w_*). This can be accomplished by using a nested analysis of variance (ANOVA), in which the variance between individuals can be divided into error variance, the variance between individuals within a species and the variance between species [18]. The variance between individuals within a species, *V_w_*, is an estimate of *E*_w_, and the variance between species, *V_b_*, is an estimate of *E_b_*+*E_c_*. Similarly we can consider the average divergence between individuals within a species, the nucleotide diversity, *π*, to be an estimate of *S_w_*, and the average divergence between individuals of different species, *d*, to be an estimate of *S_b_*+*S_c_*+*S_w_*. If we assume that *t_c_* is small relative to *t_b_*, we can ignore *E_c_* and *S_c_* and estimate the selection index as
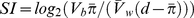
(5)where the averages are across species. If expression or sequence data is not available for both species, then we suggest that we assume that the within-species expression variance and nucleotide diversity in the species with missing data are the same as in the species for which we have data. Our method assumes that neutral sequence divergence at the locus whose expression is being analysed is an appropriate neutral standard and that *t_b_* and *t_w_* are the same for the expression and sequence data. This is likely to be the case for *cis*-acting mutations, which appear to comprise the bulk of gene regulatory mutations [37], [38]. To estimate SI for groups of genes we suggest using the average values of *V_b_*, 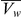, *d* and 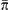 across loci; in doing this we are effectively averaging *t_b_* and *t_w_* across loci, so even if some proportion of regulatory mutations are *trans*-acting, this is unlikely to affect our estimates substantially.

When the selection index is positive, *i.e.*, when we have evidence of positive selection, we can also estimate the proportion of adaptive gene expression change, *α_e_*: If we assume that some expression mutations are advantageous then we expect *E_b_/S_b_*>*E_w_/S_w_* because advantageous mutations contribute more to divergence than they do to polymorphism. If we assume that the advantageous mutations are rare, but strongly selected, then we can ignore their contribution to polymorphism, as an advantageous mutation contributes at most twice the nucleotide diversity of a neutral mutation [39]. We then have
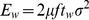
(6)and
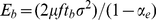
(7)where *α_e_* is the proportion of the expression divergence driven by positive selection. Hence
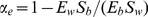
(8)or, following the same reasoning as for the selection index:
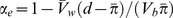
(9)This is analogous to the method for estimating the proportion of substitutions driven by positive selection [30].

### Data analysis

To estimate *V_w_* and *V_b_* from experimental data, we used a previously published expression dataset from human and chimpanzee lymphoblastoid cell lines, measured on the human-specific Affymetrix U133A microarray [40]. We masked the data by removing all probes that did not have a unique perfect match in the chimpanzee genome. Probe sets with less than four remaining probes were discarded, as smaller probe sets tend to give unreliable results [41]. Expression values were calculated with the robust microchip average (RMA) method as implemented in Bioconductor [42]–[44]. For genes with multiple probe sets on the array, we chose a single probe set at random to represent that gene.

The dataset from Choy et al. [40] included cell lines derived from 5 chimpanzees and 46 humans, of which 13 were of European descent (CEU), 19 of Han Chinese or Japanese descent (CHB/JPT) and 14 of Yoruba descent (YRI). For each human sample, two replicates were available, whereas three or four replicates were available for the chimpanzee samples. To achieve a balanced experimental design, five individuals were randomly chosen from each of the human populations, and two replicates were randomly chosen for each chimpanzee individual, so that for each analysis we had five humans and five chimpanzees with two replicates each. The between-species, within-species and error variance components were then estimated by nested ANOVA of the log-transformed expression values, with the modification that we calculated separate estimates for the human and chimpanzee within-species and error variances.

To verify that our variance estimates were unbiased even in cases with unequal variances, we used the same method to analyse simulated expression datasets that were based on the model
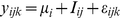
(10)where *y_ijk_* is the log_2_ expression value for species *i*, individual *j* and replicate *k*, *μ_i_* is the true mean, *I_ij_* represents individual variation and *ε_ijk_* is the measurement error. The values for *μ_I_*, *I_ij_* and *ε_ijk_* were drawn from normal distributions with variance corresponding to the between-species, within-species and error variances displayed in Table 1.

**Table 1:** Nested ANOVA estimates of variance components based on datasets with unequal variances.

	*V_b_*	*V_wh_*	*V_eh_*	*V_wc_*	*V_ec_*
Average	0.061 (0.06)	0.020 (0.02)	0.063 (0.06)	0.051 (0.05)	0.096 (0.10)
Higher *V_e_*	0.061 (0.06)	0.020 (0.02)	0.060 (0.06)	0.046 (0.05)	1.002 (1.00)
Higher *V_w_*	0.062 (0.06)	0.020 (0.02)	0.600 (0.06)	0.492 (0.50)	0.101 (0.10)
Higher *V_e_* and *V_w_*	0.062 (0.06)	0.020 (0.02)	0.060 (0.06)	0.512 (0.50)	0.995 (1.00)

*V_b_* is the between-species variance, *V_wh_* the human within-species variance, *V_eh_* the human error variance, *V_wc_* the chimpanzee within-species variance and *V_ec_* is the chimpanzee error variance. The variance estimates were averaged across 10000 simulations. The true variances used to generate the data are given in brackets. The first set of simulations was based on the average observed variances in humans and chimpanzee, and the chimpanzee error variance and within-species variances were then increased by a factor of 10.

Estimates of *π* and *d* for each gene were obtained as follows: We extracted the intron coordinates of all human autosomal protein-coding genes in Ensembl release 56 [45]. To further ensure that we were working with purely neutral sequences, we removed any sequences that were within 50 bp of a splice junction or that overlapped with exons from other genes. We also removed conserved elements identified by the phastCons program [46] by excluding all sequences that featured in the ‘Primate El’ table of the Conservation track for the human genome release hg18 in the UCSC Genome Browser [47]. The SNP frequency spectra for these neutral sequences in the CEU, CHB/JPT and YRI populations were taken from low coverage pilot data from the 1000 Genomes Project [48]. To correct for the limited power to detect very rare variants, we divided the number of observed SNPs at different frequencies by the power to detect SNPs at that frequency (estimates of detection power were kindly provided by Adam Auton). To estimate the degree of sequence divergence, we downloaded blastz alignments [49] of the human and chimpanzee genomes (releases hg18 and panTro2, respectively) from the UCSC genome browser [47], [50], [51]. We excluded sites where the human sequence was unknown (‘N’) or where the chimpanzee sequence had a quality score of 40 or below, as judged from the Quality Scores track in the UCSC Genome Browser.

In equations 5 and 9 we need to subtract the average nucleotide diversity, across our two species, from *d*. Unfortunately we do not have data from chimpanzee and so we assumed that the nucleotide diversity for each gene was the same in humans and chimpanzees. The true chimpanzee value is likely to be larger [52], [53], which means that our estimate of *d* is slightly inflated and will cause our test to be somewhat conservative. To test whether this had a major influence on our results, we repeated the analysis, assuming that the chimpanzee average heterozygosity was 10-fold larger than the one found in humans.

To gauge the accuracy of selection index estimates for individual genes, we generated datasets of 5000 genes where all genes had a true selection index of −5, −2, 0, 2 or 5. In our simulations, we drew *V_w_* from a uniform distribution ranging from 10^−4^ to 1 and used this value and the true selection index to set the true *V_b_* for that gene. Note that the results of this analysis are independent of the magnitude of *V_w_*. We then estimated *V_b_* based on two species means drawn from a normal distribution with a mean of 0 and variance corresponding to the true *V_b_*, and used this to calculate the estimated selection index.

## Results

We propose a selection index for gene expression based on the well-established McDonald-Kreitman test for sequence data [29]. Under a neutral scenario, suitably measured expression divergence is expected to increase linearly with time, just as we expect for neutral sequence evolution. We can therefore construct the index by contrasting the expression divergence between and within species to the level of neutral sequence divergence between and within species. Negative values of the selection index are indicative of stabilizing selection, whereas positive values suggest adaptive evolution. Here, we have applied the selection index to gene expression data from human and chimpanzee cell lines [40]. We chose this dataset because it contains replicate measurements from multiple individuals from both species, allowing us to remove the error variance from our estimates of between-species and within-species expression variance using nested ANOVA.

Nested ANOVA assumes that the experimental design is balanced, that the data is normally distributed and that variances do not differ between groups [54]. Before proceeding, we therefore ensured that the expression data fulfilled these requirements. The design of the original dataset was not balanced, as it contained different numbers of individuals and replicates for the two species. Although methods exist to estimate variance components based on unbalanced designs, they tend to be either cumbersome or give biased results [55]. We therefore chose to balance the design by randomly excluding some of the raw data, leaving us with five individuals and two replicates from chimpanzees and from each of the three human populations represented in the original dataset. After processing the resulting dataset (see Materials and Methods) we examined the distributions of the standardised log-transformed expression values, which in all cases proved to be approximately normal. However, using single-classification ANOVA to estimate the within-species and error variance for each gene, we found that the variances were not equal: the average human within-species variance was 0.02 while the average chimpanzee within-species variance was 0.05. The difference could be due to the fact that chimpanzees have a higher effective population size than humans do [56], [57], or because the sampled chimpanzees were bred in captivity and may therefore belong to different subspecies [58]. The mean error variance also differed between humans and chimpanzees, which might reflect variation in the establishment and maintenance of the cell lines. However, unequal variances are only problematic if they introduce bias into the nested ANOVA procedure. To test if this was the case, we simulated datasets of 10000 replicates with differing within-species and error variances, calculated the variance components using nested ANOVA and compared the estimated between-species variance to the set value (Table 1). We found that a 10-fold increase in chimpanzee within-species and error variances only had a marginal effect on the between-species variance estimate, which was overestimated by around 3%. In cases with unequal variances our test may therefore give a biased estimate of the selection index, but the overall effect is negligible.

We used intronic sequences as our neutral reference as it has previously been shown that mammalian introns are essentially neutral [59]. For these sequences we estimated the average divergence, *d*, between human and chimpanzee, as well as the nucleotide diversity, *π*, for the three human populations CEU, CHB/JPT and YRI [48]. In total, we had expression and sequence data for 7302 genes, which we used to calculate the selection index for each of the three human populations versus chimpanzee by averaging the values of *V_b_*, 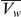, *d* and 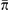 across loci and then applying equation 5 (Table 2). We constructed confidence intervals for these estimates by bootstrapping the data by gene, *i.e.*, by randomly choosing genes (with replacement) from our original data, recalculating the selection index for these new datasets and choosing the confidence limits in such a way that 2.5% of our simulated selection index values fell above the upper limit and 2.5% below the lower limit. In all cases, the selection index was significantly negative. While the estimate was somewhat higher for the CHB/JPT population, this is likely to be an artefact caused by the high error variance for these samples (Table 2), rather than a sign of varying selection pressures among human populations. Our results therefore indicate that gene expression divergence between humans and chimpanzees increases in a non-linear fashion and that stabilising selection plays a dominant role in shaping gene expression evolution even over short evolutionary distances.

**Table 2:** Calculation of the selection index for the three human populations versus chimpanzee.

Human population	*V_b_*	*V_wh_*	*V_eh_*	*V_wc_*	*V_ec_*	*d*	*π*	*SI (95% CI)*
*CEU*	6.4×10^−2^ (3.9×10^−3^)	3.7×10^−2^ (1.8×10^−3^)	4.5×10^−2^ (5.8×10^−4^)	4.7×10^−2^ (2.9×10^−3^)	1.0×10^−1^ (2.1×10^−3^)	1.2×10^−2^ (7.3×10^−5^)	6.1×10^−4^ (5.0×10^−6^)	−2.50 (−2.63, −2.37)
*CHB/JPT*	6.3×10^−2^ (3.5×10^−3^)	6.5×10^−3^ (8.9×10^−4^)	7.8×10^−2^ (9.7×10^−4^)	4.7×10^−2^ 2.9×10^−3^	1.0×10^−1^ (2.1×10^−3^)	1.2×10^−2^ (7.3×10^−5^)	5.7×10^−4^ (5.2×10^−6^)	−2.14 (−2.29, −2.00)
*YRI*	5.9×10^−2^ (3.5×10^−3^)	3.3×10^−2^ (1.6×10^−3^)	4.7×10^−2^ (9.0×10^−4^)	4.7×10^−2^ (2.9×10^−3^)	1.0×10^−1^ (2.1×10^−3^)	1.2×10^−2^ (7.3×10^−5^)	7.7×10^−4^ (5.2×10^−6^)	−2.30 (−2.43, −2.16)

Average values, with standard errors in brackets, for 7302 genes. *V_b_* is the between-species expression variance, *V_wh_* the within-human variance, *V_eh_* the human error variance, *V_wc_* the within-chimpanzee variance, *V_ec_* is the chimpanzee error variance, *d* the human-chimpanzee sequence divergence and *π* the average heterozygosity within the human population. The 95% confidence interval for the selection index (SI) was obtained by bootstrapping across genes.

**Figure 2:**
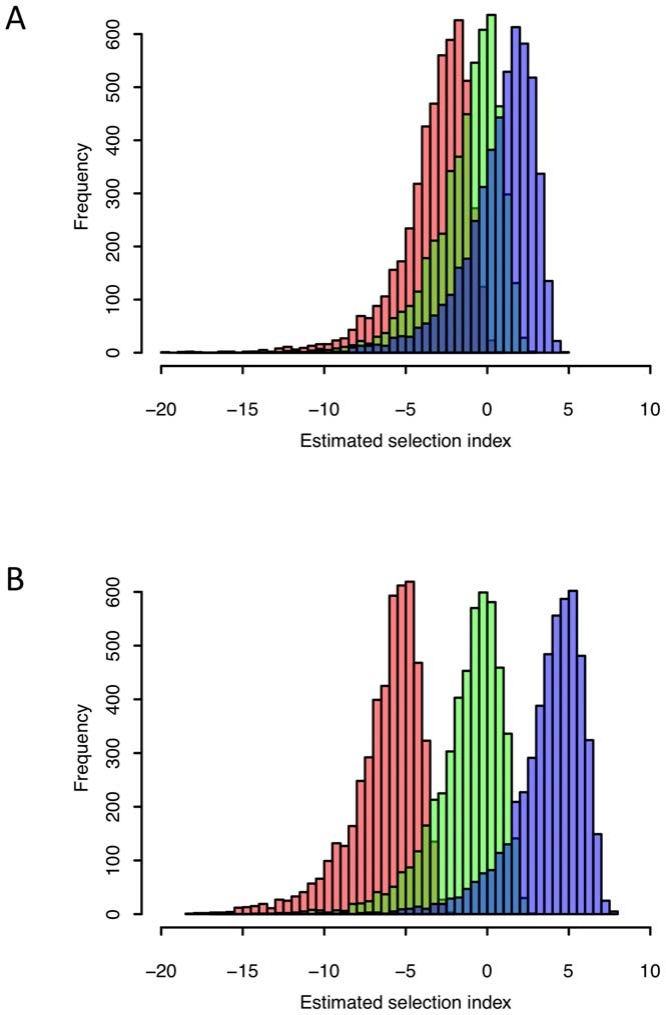
Estimates of the selection index for individual genes under different evolutionary scenarios, assuming that all measurements are without error and can be obtained from an infinite number of individuals. A. Genes with true SI = −2 (negative selection) in red, genes with true SI = 0 (neutral evolution) in green and genes with true SI = 2 (positive selection) in blue. B. Genes with true SI = −5 in red, true SI = 0 in green and true SI = 5 in blue.

Even though this dataset does not fulfil the requirements for estimation of the proportion of adaptive evolution, *α_e_*, we may still ask whether, in spite of the overarching trend of strong stabilising selection, we can use the selection index to identify adaptively evolving genes. In principle, a positive estimate of the selection index for a single gene can be taken as an indication of positive selection. To evaluate the performance of this method, we investigated the distribution of gene-specific estimates of the selection index under different evolutionary scenarios, by considering an ideal experiment where both gene expression and sequence variation could be measured without error for an infinite number of individuals. Under these conditions, any discrepancy between the true and the estimated value of the selection index will stem from the estimation of the between-species expression variance based on the two species means. As shown in Figure 2 there is considerable overlap between the distributions of selection index estimates for positively and negatively selected genes, even when all experimental error is removed. This illustrates an important difference between the evolution of gene expression and the evolution of DNA sequences: While for each gene we can base our estimate of sequence divergence on multiple sites, we only have a single measure of gene expression divergence. We therefore recommend the use of the selection index as a straightforward method to capture the main evolutionary trends for larger groups of genes, but caution against its use on a single-gene basis. Simulations such as those that we have presented here can be a valuable tool to assess the performance of the selection index in different conditions and could also be extended to include parameters for experimental error and sample size to fit a particular experimental setup.

## Discussion

The gene expression selection index encapsulates the main selective forces that affect gene expression levels in two species. It complements previous approaches that require multiple species comparisons to draw conclusions about evolutionary trends [17], [25], [28]. Our method has some similarities to the test of selection developed by Lemos et al. [22], but we infer the rate of neutral gene expression evolution from sequence data, rather than from a combination of estimates of divergence times, generation times and the typical range of mutational heritability for phenotypic characters. Furthermore, as the analysed expression and sequence data come from the same set of genes, we reduce the problem of sampling the neutral standard from a different genomic region to that in which regulatory changes are probably occurring.

In our analysis, we have made the assumption that all regulatory mutations have taken place in *cis* rather than *trans*. We believe that this is a reasonable simplification, based on experimental evidence suggesting that *cis*-regulatory effects are more common [37], [38]. However, with a more complete knowledge of the regulatory structure of different genomes, it will be possible to further refine our model to also take *trans*-regulatory mutations into account. For example, if it is known that the change in expression of a given gene is primarily due to a specific regulatory factor that operates in *trans*, it might be more appropriate to base the neutral expectation on sequences from the *trans* factor locus. However we note that our method is most useful when applied to a set of genes, meaning that *d* and 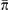 are estimates of the genome-wide values. Hence, our estimate of the selection index will be unbiased unless the genes responsible for *trans* changes have unusual values of *d* and *π*.

Our estimates of the selection index for human and chimpanzee lymphoblastoid cell lines suggest prevalent stabilising selection on gene expression levels. While this contradicts some early estimates [25], [60], it is in line with later analyses of primate gene expression [17], [22]. Thus our study reinforces the view that gene expression evolution is constrained by negative selection even over relatively short time spans.

To what extent are lymphoblastoid cell lines a suitable system to study gene expression evolution? It is known that many genes are differentially expressed between these cell lines and the cells from which they were originally derived, although the magnitude of change tends to be minor [61]. On the other hand, the use of cell lines that can be grown under control conditions has some potential advantages over tissue samples, where it is often not possible to match individuals with regard to environmental factors have been found to influence gene expression [62], [63]. Another question is whether lymphoblastoid cell lines are representative of the entire organism, as the selection index will vary between tissues, cell types and developmental stages. While our results are consistent with analyses of brain and liver from adult humans and chimpanzees [17], [22], we cannot exclude that an equivalent analysis of other samples could lead to different conclusions. We do however note that lymphoblastoid cell lines are derived from blood cells involved in the body's immune response and that genes with functions in immunity show signs of positive selection on both protein-coding and non-coding sequences [11]. We therefore do not have any reason to believe that these cell lines should be particularly void of adaptive changes in gene expression, which could cause the selection index to be exceptionally low. We therefore consider it very likely that strong stabilising selection is a general feature of human and chimpanzee gene expression evolution.

Negative estimates of the selection index do not necessarily imply that the species under study have not experienced adaptive evolution of gene expression, as positive selection acting on a few genes might be overshadowed by negative selection acting on others. The extent to which human gene expression evolution has been adaptive is however a question that remains to be settled: Lemos et al. [22] did not identify any targets of positive selection in human and chimpanzee brain and liver, while Kudaravalli et al. [8] estimated that 0.1% of human genes had experienced very recent positive selection, as judged from lymphoblastoid cell lines from the YRI population. Contrary to this, Brawand et al. [64] identified a number of candidates for positive selection based on their analysis of gene expression in six tissues. The difference in sensitivity between these analyses might to some extent be explained by the use of different null hypotheses: Lemos et al. [22] assumed that the between-species variance accumulated in a linear fashion, while Brawand et al. [64] used a model that incorporated strong stabilising selection. Our results indicate that this latter model is preferable for humans and chimpanzees, even though they diverged relatively recently.

When the selection index is positive, it is possible to calculate the proportion of the between-species expression variance that is contributed by adaptive evolution, *α_e_*. This estimate is likely to be conservative as some genes may be constrained by stabilising selection. Assuming constant population size, a value of *α_e_* that is significantly above 0 is therefore powerful evidence of the role of positive selection. While human-chimpanzee comparisons do not currently lend themselves to this type of analysis, it would be interesting to investigate gene expression evolution within the *Drosophila* genus, as some of the species may be closely enough related for gene expression divergence to increase relatively linearly [28] and positive selection on protein-coding sequences has played a much larger role in *Drosophila* than in mammals [31], [65]. Following the method of Eyre-Walker and Keightley [66] it might also be possible to determine the distribution of fitness effects for mutations that affect gene expression and use this information to control for the effects of slightly deleterious mutations that contribute to within-species but not between-species expression variance, thereby making it possible to calculate *α_e_* for a wider range of species, including humans and chimpanzees.
